# Clopidogrel resistance "Live" – the risk of stent thrombosis should be evaluated before procedures

**DOI:** 10.1186/1477-9560-7-6

**Published:** 2009-05-19

**Authors:** Zuzana Motovska, Petr Widimsky, Iuri Marinov, Robert Petr, Jaroslava Hajkova, Jan Kvasnicka, the PRAGUE-8 study Investigators

**Affiliations:** 1 Third Medical Faculty Charles University & University Hospital Kralovske Vinohrady, Prague, Czech Republic; 2 The Institute of Hematology and Blood Transfusion, Prague, Czech Republic; 3 First Medical Faculty Charles University and Thrombotic Centre General University Hospital, Prague, Czech Republic

## Abstract

Every year, millions of people undergo percutaneous coronary intervention (PCI) with intracoronary stent implantation. A patient from the PRAGUE-8 trial (Optimal pre-PCI clopidogrel loading: 600 mg before every coronary angiography vs. 600 mg in the cath-lab only for PCI patients) is described who suffered from acute stent thrombosis. This patient did not have any relevant inhibition of platelet activation even after the 600 mg dose of clopidogrel. Dose uptitration would have been ineffective. New P2Y_12 _receptor inhibitors are desperately needed. In the light of recently published data, the use of prasugrel may be considered as an alternative.

## Introduction

Every year, millions of people undergo percutaneous coronary intervention (PCI) with intracoronary stent implantation. Dual antiplatelet therapy – aspirin plus clopidogrel – is recommended for the reduction of acute and subacute stent thrombosis [1,2]. Despite combined antiplatelet therapy, stent thrombosis persists at a rate of 0.5–2% in elective cases, and up to 6% in patients with acute coronary syndromes [3]. Stent thrombosis is a life-threatening event [4]. In addition, also in cases of immediate reperfusion therapy by means of emergency PCI, patients with stent thrombosis have developed a major myocardial infarction, with consequent significant decline in left ventricular function – a strong negative predictor of long-term survival [3]. "Retrospective" laboratory testing in patients with stent thrombosis has shown that poor response ("resistance") to antiplatelet therapy is a risk factor for this event [5-7].

## Case report

A 67-year old woman was admitted to Cardiocentre for an elective coronary angiography, because of changes on the ECG (new negative T waves in leads I, aVL, V1-V3) and new anteroapical hypokinesis seen by echocardiography. She was a cigarette smoker, with a history of diabetes, hypertension, hypercholestrolemia on statin therapy (atorvastatin), and with known coronary artery disease on aspirin. The patient fulfilled the inclusion criteria of the PRAGUE-8 trial (see section methods) [8]. After signing of informed consent, she was randomized into group B of this study, and also participated in the vasodilator stimulated phosphoprotein (VASP) phosphorylation state and genetic laboratory substudies. In the laboratory substudy, the time course of platelet inhibition after clopidogrel (600 mg loading dose followed by 75 mg per day) was investigated.

**Figure 1:**
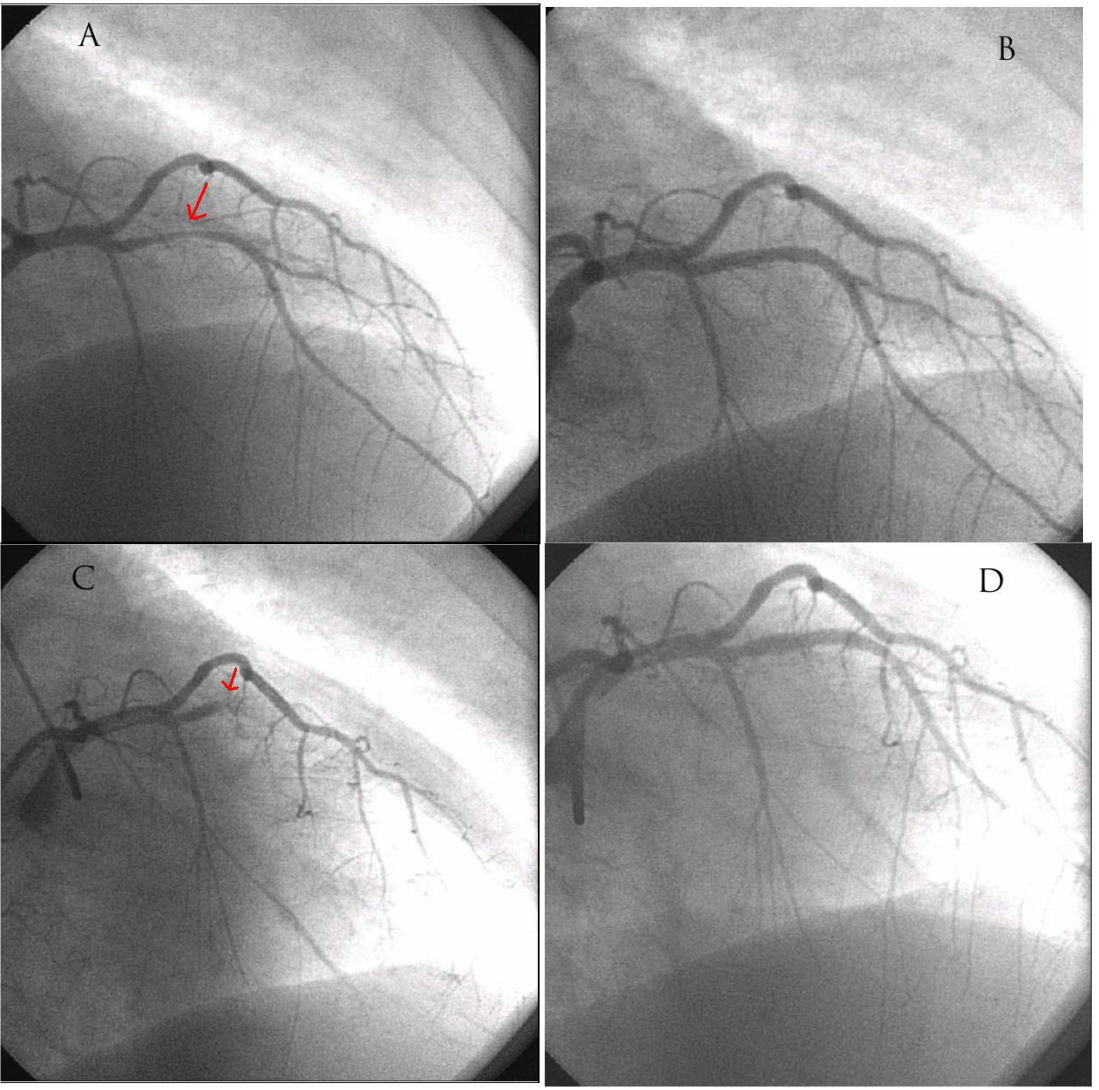
**Coronary angiography and pecutaneous coronary intevention studies**. A, B – elective during the first hospitalization; C, D – urgent during the second hospitlization.

On the second day of hospitalization, the patient underwent a coronary angiography, which showed an 80% stenotic lesion on her left anterior descending artery. The lesion was treated with ad hoc performed PCI with the implantation of a bare metal stent. The success of the procedure was optimal (Figure 1A, B). The next day, the patient was stable, did not have any complications, and was discharged home. The recommendation for drug therapy was as follows: ASA (100 mg/d), clopidogrel (75 mg/d), metoprolol, ramipril, atorvastatin, peroral antidiabetic.

**Figure 2:**
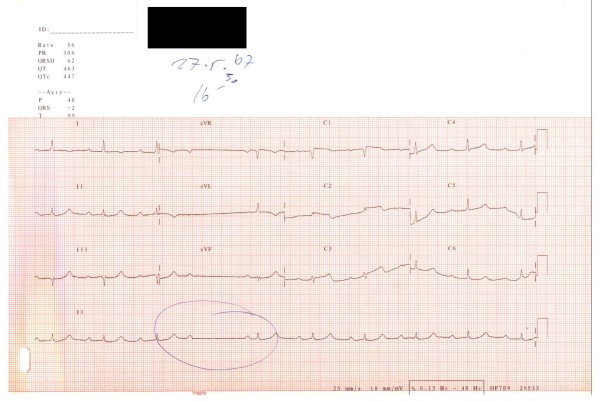
**ECG at the second hospital admission**.

46 hours after stent implantation, the patient returned to the hospital because of chest pain, vertigo and swelling. There were ST segment elevations in leads V1-V3 and a new second-degree A-V block according to the ECG (Figure 2). An emergency coronary angiography was performed, and showed 100% occlusion of the left anterior descending artery due to acute stent thrombosis. Immediate ballon angioplasty with heparin and eptifibatide opened the artery and led to a good angiographic result (Figure 1C, D).

**Figure 3:**
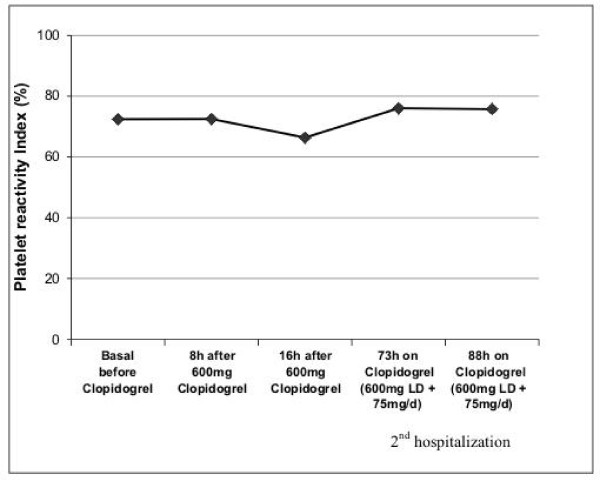
**Clopidogrel efficacy; ADP-induced platelet activation (Platelet reactivity index) **[11]**before and after clopidogrel**.

What did the VASP phosphorylation study show? There was no reaction to the administration of clopidogrel – the patient was completely "resistant" to this drug (Figure 3). Interestingly, by the second admission the ADP-stimulated platelet reactivity was even higher than the basal value without clopidogrel therapy. The most probable explanation for this was an acute myocardial infarction, which was the reason for the second hospitalization.

In the genetic substudy we investigated the prevalence of nine platelet and haemostatic gene polymorphisms. The results of this extensive genetic testing are shown in Table 1. Detected single nucleotid polymorphisms of P2Y_12 _and GPIIIa receptors had been recognized as possible intrinsic mechanisms of clopidogrel resistance [8,9].

**Table 1:** Genetic testing for platelet polymorphisms and procoagulation state

**Polymorphism**	**Result**
Leiden mutation	Negative

Factor II mutation	Negative

P2Y12 H1/H2 haplotyp	Negative

P2Y12 (32C/T)	Heterozygote

GPVI (13254C/T)	Negative

PAR-1 (IVSn-14A/T)	Heterozygote

GPIIIa (PlA1/PlA2)	Heterozygote

COX-1 (-842A/G)	Negative

COX-1 (50C/T)	Negative

## Methods

***The PRAGUE-8 study ***was a randomized multi-center open label clinical trial which compared the routine clopidogrel 600 mg pretreatment more than 6 hours before coronary angiography (group A) with the selective administration of clopidogrel 600 mg only for PCI patients in the catheterization laboratory, after the coronary angiography and just prior to PCI (group B) (ClinicalTrials.gov identifier NCT00432120) [10]. The inclusion criteria were: a planned elective coronary angiography for suspected or proven chronic stable coronary artery disease or medically fully stabilized acute coronary syndrome, a signed written informed consent, and age ≥ 18 years. The primary end point was the first clinical occurrence of any of the following: death, periprocedural myocardial infarction, stroke or transient ischemic attack, or re-intervention within 7 days. Secondary end-points were: periprocedural troponin elevation (> 3 × ULN), TIMI-flow after PCI, bleeding complications, and each individual component of the combined primary end-point.

***Vasodilator Stimulated Phosphoprotein (VASP) ***is a platelet intracellular actin regulatory protein. The phosphorylation of VASP is regulated by the cyclic adenosine monophosphate cascade. Prostaglandin E_1 _activates this cascade, whereas it is inhibited by adenosine diphosphate through P2Y_12 _receptors. The phosphorylation status of VASP correlates with P2Y_12 _receptor inhibition, whereas its non-phosphorylation state correlates with the active form of the P2Y_12 _receptor. Levels of VASP phosphorylation/dephosphorylation reflect P2Y_12 _inhibition/activation [11]. The effect of thienopyridines can be demonstrated by the persistence of VASP phosphorylation induced by prostaglandin E_1_, even with the simultaneous addition of ADP [12,13].

***Analysis of VASP phosphorylation by flow cytometry***. Flow cytometry analysis was done in a laboratory awarded by the Joint Commission International accreditation. Examiners analyzing the VASP phosphorylation state were blinded to the patient-related data. The VASP phosphorylation state was determined in whole blood by flow cytometry using the standardized (CE, IVD) platelet VASP/P2Y_12 _assay (Biocytex, France). Briefly, the blood sample was first incubated with prostaglandin E_1 _alone or prostaglandin E_1 _+ ADP. After cellular permeabilization, VASP under its phosphorylated state was labeled by indirect, no-wash immunofluorescence using a specific monoclonal antibody (16C2). Dual-color flow cytometric analysis was performed on a Becton Dickinson FACS Calibur cytometer to compare the two tested conditions, and to evaluate the capacity of ADP to inhibit VASP phosphorylation for each sample. The platelet population was identified by forward scatter, side scatter and fluorescence (anti CD61-PE) gating, then 5000 events were acquired at low rate to list mode files.

A platelet reactivity index (PRI) was calculated from the median fluorescence intensity (MFI) of samples incubated with prostaglandin E_1 _or prostaglandin E_1 _and ADP, according to the formula: PRI = [(MFI_(PGE1) _- MFI_(PGE1+ADP)_/MFI_(PGE1)_] × 100. If inhibition of the platelet P2Y_12 _receptor by clopidogrel occurs, ADP fails to cause a decrease in VASP phosphorylation and the PRI converges to 0. In contrast, if treatment is ineffective (i.e. a low responder), dephosphorylation of VASP by ADP still occurs, and the PRI reaches values of ≥ 0.5 or greater [11].

## Discussion

How should this patient be managed? What do the recommendations say? We have only the expert consensus position regarding aspirin resistance [14]. There is no official expert statement on the problem of clopidogrel ineffectiveness or resistance. The ACC/AHA guidelines recommends that "in patients in whom subacute thrombosis may be catastrophic or lethal, platelet aggregation studies may be considered and the dose of clopidogrel increased to 150 mg per day if less than 50% inhibition of platelet aggregation is demonstrated" [2].

As Figure 3 shows, our patient did not have any relevant inhibition of ADP-induced platelet reactivity even after the 600 mg clopidogrel. Therefore, dose up-titration would have been ineffective. Administration of GPIIb/IIIa inhibitor during emergency PCI for stent thrombosis has been identified as the only independent predictor for the prevention of recurrent stent thrombosis [3]. Third generation tienopyridine prasugrel provides faster onset and greater inhibition of P2Y_12 _receptor-mediated platelet aggregation than clopidogrel, because of greater and more efficient generation of the active metabolite [15,16]. Pharmacodynamic data have also shown that prasugrel achieves a sufficient degree of platelet inhibition within 30 minutes after treatment [14]. In a crossover study, non-responders to 300 mg of clopidogrel responded effectively to a loading dose of 60 mg of prasugrel [17]. This favorable laboratory profile resulted in clinical efficacy for prasugrel. The TRITON-TIMI 38 [18] trial recently showed that in comparison to clopidogrel, prasugrel therapy was associated with significantly reduced rates of ischemic events in patients with acute coronary syndromes with scheduled PCI. Stent thrombosis was reduced by approximately 50% in the prasugrel group compared to the clopidogrel group. The price for this clinical benefit was a significantly higher risk of major bleeding.

In patients with planned elective PCI for whom the optimal time for clopidogrel pretreatment is a day before procedure [19], a test for drug effectiveness before the procedure will identify patients with insufficient platelet inhibition. In these patients, the administration of GPIIb/IIIa inhibitors during the procedure and a higher dose (150 mg per day) of clopidogrel after stent implantation should be advised to prevent stent thrombosis [2,3]. In patients with no response to the clopidogrel loading dose, prasugrel seems to be an effective alternative [15-18,20]. Flow cytometric determination of VASP phosphorylation state strongly correlated with the inhibition of ADP-induced platelet aggregation resulting from specific P2Y_12 _blockade by a specific antagonist of the P2Y_12 _receptor (AR-C69931MX) [6]. This test gives reliable results in whole blood samples kept at room temperature for up to 48 h [6,21]. Aspirin and abciximab did not interfere with VASP phosphorylation [9,19]. A flow cytometric analysis of VASP phosphorylation can be recommended as an index (the gold standard) of the efficacy of P2Y_12 _inhibitors [21].

## Conclusion

In patients with planned elective PCI a flow cytometric VASP phosphorylation test will identify those without sufficient P2Y_12 _receptors inhibition and with risk of stent thrombosis. In patients with no response to the clopidogrel loading dose, prasugrel seems to be an effective alternative.

## Competing interests

The authors declare that they have no competing interests.

## Authors' contributions

ZM was the co-principal investigator of the PRAGUE-8 trial, participated in the design of the study and coordination, drafted the manuscript. PW was the co-principal investigator of the PRAGUE-8 trial, participated in the design of the study and coordination, participated in critical revision of the manuscript for important intellectual content. IM carried out the flow cytometric analyses. RP carried out the administrative, technical, or material support. JH carried out the molecular genetic studies. JK carried out the molecular genetic studies. All authors read and approved the final manuscript.
